# Artificial intelligence in lung cancer diagnostic imaging: a review of the reporting and conduct of research published 2018–2019

**DOI:** 10.1259/bjro.20220033

**Published:** 2023-06-06

**Authors:** Patricia Logullo, Angela MacCarthy, Paula Dhiman, Shona Kirtley, Jie Ma, Garrett Bullock, Gary S. Collins

**Affiliations:** 1 Centre for Statistics in Medicine, Nuffield Department of Orthopaedics and Musculoskeletal Sciences, University of Oxford, Oxford, United Kingdom; 2 NIHR Oxford Biomedical Research Centre, John Radcliffe Hospital, Oxford, United Kingdom; 3 UK EQUATOR Centre, Nuffield Department of Orthopaedics and Musculoskeletal Sciences, University of Oxford, Oxford, United Kingdom; 4 Department of Orthopaedic Surgery, Wake Forest School of Medicine, Winston-Salem, North Carolina, United States

## Abstract

**Objective:**

This study aimed to describe the methodologies used to develop and evaluate models that use artificial intelligence (AI) to analyse lung images in order to detect, segment (outline borders of), or classify pulmonary nodules as benign or malignant.

**Methods:**

In October 2019, we systematically searched the literature for original studies published between 2018 and 2019 that described prediction models using AI to evaluate human pulmonary nodules on diagnostic chest images. Two evaluators independently extracted information from studies, such as study aims, sample size, AI type, patient characteristics, and performance. We summarised data descriptively.

**Results:**

The review included 153 studies: 136 (89%) development-only studies, 12 (8%) development and validation, and 5 (3%) validation-only. CT scans were the most common type of image type used (83%), often acquired from public databases (58%). Eight studies (5%) compared model outputs with biopsy results. 41 studies (26.8%) reported patient characteristics. The models were based on different units of analysis, such as patients, images, nodules, or image slices or patches.

**Conclusion:**

The methods used to develop and evaluate prediction models using AI to detect, segment, or classify pulmonary nodules in medical imaging vary, are poorly reported, and therefore difficult to evaluate. Transparent and complete reporting of methods, results and code would fill the gaps in information we observed in the study publications.

**Advances in knowledge:**

We reviewed the methodology of AI models detecting nodules on lung images and found that the models were poorly reported and had no description of patient characteristics, with just a few comparing models’ outputs with biopsies results. When lung biopsy is not available, lung-RADS could help standardise the comparisons between the human radiologist and the machine. The field of radiology should not give up principles from the diagnostic accuracy studies, such as the choice for the correct ground truth, just because AI is used. Clear and complete reporting of the reference standard used would help radiologists trust in the performance that AI models claim to have. This review presents clear recommendations about the essential methodological aspects of diagnostic models that should be incorporated in studies using AI to help detect or segmentate lung nodules. The manuscript also reinforces the need for more complete and transparent reporting, which can be helped using the recommended reporting guidelines.

## Introduction

Survival from lung cancer is still poor worldwide: less than 20% of patients survive for 5 years^1,2^ and lung cancer is the leading cause of cancer death globally.^2^ Diagnosing cancer early offers more treatment options and the possibility of longer survival.^1,3^

The human eye can easily detect lesions measuring >30 mm in diameter on a chest X-ray or a CT scan. These larger lesions are considered indicative of cancer, warranting a biopsy for diagnosis. However, lung nodules, or lesions measuring <30 mm, are harder to detect and investigate further.^3^

The lesion size, although highly indicative,^3,4^ is not the only factor predicting malignancy—and certainly not the only challenging feature seen on lung scans.^5^ When looking at a lung lesion on a CT scan or X-ray, several other physical characteristics of the nodule need to be considered by the radiologist, such as location, shape, texture (solid or partially solid), and the presence of calcification, all having clinical implications. Non-solid or subsolid nodules, particularly “ground-glass nodules” (GGNs), are difficult to interpret as benign or malignant based on imaging only. Most GGNs are small, exhibit lower contrast and have less well-defined borders than solid nodules, making it possible for them to be missed by radiologists.^6^ This type of opacity on the scans, described as a “nodular shadow”, has been detected more frequently because of the increased use of CT for lung cancer screening.^3,7^ GGNs are usually associated with the early stages of adenocarcinoma of the lung—but can also represent inflammation, fibrosis or benign hyperplasias.^7,8^ The differentiation between malignant and benign nodules can be complex, and the ability to assess a large number of visual features simultaneously and their relation to specific outputs (*e.g.* malignancy) could be helpful.^9,10^

Harnessing emerging technologies could help radiologists to detect suspicious lesions and possibly reduce their workload by doing it faster. Radiomics, as highlighted by the World Health Organization (WHO), is an emerging technology that seeks to extract meaningful features from imaging data, possibly helping to identify lung nodules with malignant potential.^1^ Radiomics involves the extraction of a large number of image features from radiology scans—features that may not be detectable by the human eye.^1,11^ Artificial intelligence (AI) goes beyond detecting certain features from images by aiding decision making.

AI has been increasingly tested in several fields of human health,^9,11–15^ including pulmonary cancer,^10,16–19^ speeding up diagnosis, which could, in principle, save lives by the delivery of healthcare sooner.^20^ It has been applied to imaging of the chest, breast, brain, abdomen, pelvis, and musculoskeletal system,^21^ and there has been a substantial increase in published manuscripts on the use of this technology.^13^

The availability of local or shared public repositories of images from clinical trials, screening initiatives or materials from public contests for AI development (called challenges) has facilitated research into the application of AI in lung cancer imaging.^10,16^ However, data-driven modelling such as AI and machine learning (ML) may not necessarily translate into a clinical application because of poor study methodology and poor reporting.^22,23^ The methodology of prediction model studies using AI or ML specifically for the detection or diagnosis of lung cancer has not been evaluated so far.

### Study objective

The objective of this study is to describe the methodologies used to develop and evaluate cancer prediction models that use AI to detect, segment (delineate borders), or classify pulmonary nodules as benign or malignant.

## Methods

### Study design and literature search

This review, conducted at the Centre for Statistics in Medicine (CSM), Nuffield Department of Orthopaedics and Musculoskeletal Sciences, University of Oxford, was based on published study reports and did not involve patient data. No ethical approval was required.

On 28 October 2019, we searched for relevant articles published in journals indexed in the MEDLINE and EMBASE databases via OVID. With the help of an information specialist (SK), we developed sensitive search strategies for each database because of the considerable variation in terminology for AI, ML, and the rapidly emerging naming conventions for algorithms being developed and implemented by researchers, which have implications for indexing and retrieval.

The search strategies included both controlled vocabulary headings (*e.g.* MeSH and EMTREE) and free-text search terms (searched in the title, abstract or keyword fields) for three main search facets covering AI or ML terms (*e.g.* ‘deep learning’, ‘machine learning’ and ‘artificial intelligence’), imaging terms (*e.g.* ‘ultrasonography’, ‘diagnostic imaging’, ‘magnetic resonance imaging’ and ‘computed tomography imaging’) and terms related to lung cancer (*e.g.* ‘pulmonary nodule’, ‘lung tumour’, ‘respiratory tract cancer’, ‘lung carcinoma’ and ‘endobronchial lesion’). All three search facets were combined with ‘AND’. To ensure a contemporary sample of research studies, we applied a publication date limit of 2018–2019. For the EMBASE search, we additionally applied a limit to exclude conference abstracts from the search results. The full search strategies for each database are provided in Supplementary Material 1.

### Study eligibility criteria

We included studies reporting development only, development with external validation, and external validation only of a prediction model using any type of AI for the detection, segmentation, or classification of pulmonary nodules as benign or malignant using diagnostic imaging of the human lung. We defined imaging examination studies as those involving CT scans, X-rays, MRI, positron-emission tomography (PET-CT), diagnostic ultrasound, or bronchoscopy.

As it is difficult to find universally accepted definitions of AI,^12^ it was here defined as any type of computerised system that performed ‘tasks’ typically requiring human intelligence or the ability to make decisions; or a computer algorithm that learns from data, identifies patterns and makes diagnostic predictions (See Box 1 for the working definitions in this study).^10,14,24,25^ We considered studies using ML and any computerised algorithm that solves problems using rules, improving automatically through experience or multiple iterations.

We excluded studies on mesothelioma, mediastinal tumours, and metastatic lesions in organs other than the lung. We also excluded studies developing or evaluating prediction models for outcomes based on survival, clinical trials about therapeutic or educational interventions, and studies about analyses of language used in medical records. We excluded studies of the imaging of lung pathology specimens, and studies that developed or used synthetic models resembling the human body (phantoms).We excluded articles not published or available in the English language,secondary studies (*e.g.* reviews and commentaries), and study protocols.

Box 1. **Working definitions of terms used to describe methods in the reviewed studies**

**Artificial intelligence (AI)**: An overarching term referring to the capability of a computer program or system to reproduce the human capacity of learning, performing tasks, and applying decision rules.^20,24^ In healthcare, these tasks may be detecting or diagnosing a health condition or making predictions about the evolution of disease (prognosis). AI can include machine learning, deep learning, neural networks, convolutional neural networks, and other types of architectures.^20,24^ A machine-learning algorithm continuously updates itself, “learning” more to improve task performance. Deep learning uses layered structured algorithms that require larger data sets for training.^10,14,25,26^ “Shallow” learning includes decision trees, support vector machines and random forests.^26^

**Classification**: AI is often used to classify a finding in an image as benign or malignant. Classification tools consider the shape, texture, frequency of occurrence, and overall features of the lesion.

**Cross-validation**: Cross-validation uses different proportions of the available data to train, validate, or test a model on different iterations (k). A k-fold validation will randomly split a data set into k-folds and use k-1 folds to develop a model and the remaining fold to validate it. This process is repeated k-times, producing k-sets of model testing results. For example, in 10-fold cross-validation, 90% (9-folds) of the data is used to develop the model and 10% (1-fold) is used to test it. This procedure is repeated until all 10 folds have been used for development and testing, producing 10 sets of model performance results which are then averaged.

**Detection**: Some AI tools aim to detect lesions or nodules without classifying or applying diagnostic criteria to them. Studies develop these tools for use in lung cancer screening programs, to detect small lesions that need further clinical investigation, or to enhance identification of lesions seen by radiologists.

**Ground**-**glass lesion**: A nodule with poorly defined borders, with a hazy format or blurred edges.

**Hyperparameter tuning**: The parameters of the AI modelling method that control the learning process (hyperparameters) used to develop the model, are optimised or tuned. This process is also sometime referred to as ‘model validation’ in AI modelling.

**Model development (training)**: The process of developing a model using AI modelling methods. An AI algorithm (*e.g.,* neural network) is applied to a development (training) data set, where it learns from the data and creates a prediction model.

**Model validation (testing)**: After a model has been developed and tuned, it is tested and its performance evaluated. Testing a model at this stage is synonymous with internal or external validation of statistically developed prediction models.

**Segmentation**: The delineation or definition of the borders or limits of general anatomical structures in an image, of which a lesion is an example. It can be done in two or three dimensions, such as when measuring a nodule’s volume. Segmentation can be the final objective of the study or be performed as a pre-processing step in studies aiming to do something else, such as classifying a lesion as benign or malignant. It can be carried out manually by radiologists or by using AI tools. Some studies compare the results of human and machine segmentation.

### Study selection

We imported all references retrieved by the search into Rayyan QCRI,^27^ where we excluded duplicate references manually and also using the Rayyan automatic deduplication function. A single reviewer (PL) assessed the eligibility of each article based on the title and abstract with reference to the review eligibility criteria. Next, two independent reviewers (PL and AM) conducted the full-text screening. Where necessary, a third reviewer (SK) adjudicated on disagreements. Two independent statistical reviewers (PD and JM) reviewed the final sets to ensure the correct inclusion of eligible studies.

A random list of identification numbers was generated and assigned to the included studies using Microsoft Excel. Studies were randomly allocated to three reviewer pairs for independent and double data extraction (JM/PL, GB/PL, PD/AM). Conflicts in the extracted data were resolved within each pair of data extractors.

### Data extraction form and items collected

The data extraction form was created using the OnlineSurveys platform.^28^ We first established a glossary of technical terms to be consulted by the extractors before extracting any data. We then defined a list of questions and their responses as multiple choice or free-text boxes. The group reviewed the questions and their answer options calling upon their experience in prediction modelling, oncology, and imaging, and considering: (i) issues in the reporting of diagnostic models,^12,23,29^ such as the definition of the study as being about developing or validating a tool, and the type of imaging technique and algorithm used; and (ii) topics seen during screening.

We piloted the data extraction form on five studies with different study designs. The extractors discussed the pilot results to ensure consistent data extraction and amended the data extraction form accordingly.

The final data extraction form included questions on study information, funding, study aims, image types, details of imaging pre-processing, study type, data sources, the sample size used for development and validation, AI model type, model validation methods, patient characteristics, model performance measures and reporting guideline use. The data extraction form is provided in Supplementary Material 2.

### Data analysis

Data are summarised using descriptive statistics. The sample size and analyses used in studies were described using median, interquartile range (IQR), and range. Here, we describe the number of patients, images, and nodules used for model training and validation, where applicable, and report the total sample size and number of events. Data were exported to STATA v. 15 where they were ‘cleaned’ and analysed.^30^

## Results

The search retrieved 5238 references, 3271 of which remained after de-duplication. Title and abstract screening excluded 3032 studies. Another 15 studies were excluded as their full text was not available, and 7 studies were excluded as their final publication date was outside the 2018–2019 date range used in the search.

The full texts of 217 studies were screened. From these, 64 were excluded (25 did not aim to detect, segment, or classify lung cancer; 12 used phantoms; and 27 did not use AI). We included 153 studies in our review (listed in Supplementary Material 3). A flowchart of studies included in the review is provided in Supplementary Material 4.

### Study characteristics

Of the 153 included studies, most were model development-only studies (*n* = 136/153, 88.9%), and 12 (7.8%) were both developing and validating models. Just five studies (3.2%) were validating-only models.

Over half of the studies (*n* = 79/153, 51.6%) aimed to classify images of nodules as benign or malignant, 45.1% (*n* = 69) aimed to detect lung nodules, and 22.9% (*n* = 35) aimed to segment lung images (Table 1). A public database of images was used in 57.5% of studies (*n* = 88/152 studies reporting the image source). CT scans of the lung were the most common type of image used (*n* = 127/153, 83%). Supplementary Material 5 shows the descriptions of the lung image databases used in the studies reviewed. Of the studies working on a data set with a name, the source used the most was the public database LIDC-IDRI (Lung Image Database Consortium-Image Database Resource Initiative). Patient characteristics were reported in 41 studies (26.8%), of which age and sex were the most common (*n* = 38/41, 92.7% and *n* = 40/41, 97.6%, respectively).

**Table 1.** Overall characteristics of the included studies (*n* = 153)

Study characteristics	n (%)
**Aim** ^a^	
Classification	79 (51.6)
Detection	69 (45.1)
Segmentation	35 (22.9)
Prediction	4 (2.6)
Improvement of image quality	4 (2.6)
Feature extraction, diagnostic performance of machine learning	2 (1.3)
**Image type used**a	
CT	127 (83)
Low-dose CT	13 (8.5)
X-rays (radiographs of the chest)	11 (7.2)
PET-CT	10 (6.5)
Ultrasound/echography/sonogram	2 (1.3)
MRI	1 (0.7)
Bronchoscopy (video allowing direct visualisation)	1 (0.7)
**Data source type reported**	**152** (**99.3**)
Public database	88 (57.5)
Hospital or research institution	39 (25.5)
Mixed public database and hospital	12 (7.8)
Other mixed sources	5 (3.3)
Trial	8 (5.2)
**Data source used**	**153** (**100**)
Did not use data sets	50 (32.7)
LIDC	55 (35.9)
LUNA	18 (11.8)
LIDC plus LUNA	2 (1.3)
Other individual sources	18 (11.8)
Other mixed sources	9 (5.9)
Unclear source	1 (0.7)
**Reported patient demographics**	**41** (**26.8**)
Sex	38 (92.7)
Age	40 (97.6)
Smoking status	14 (34.1)
Race or ethnicity	5 (12.2)
Personal medical history	3 (7.3)
Body weight	2 (4.9)
BMI	2 (4.9)
Family history	1 (2.4)
**Number of models developed and reported**	**148** (**100**)
One model	103
Two models	10
Three models	14
Four models	7
Five models	7
Six models	3
Seven models	2
Eight models	2

BMI, body mass index; LIDC, Lung Image Database Consortium; LUNA, lung nodule analysis; PET, positron emission tomography.

a More than one alternative possible.

One study cited the guideline for reporting multivariable prediction models for individual prognosis or diagnosis (TRIPOD).^31^ Another study cited STARD for reporting diagnostic accuracy studies.^32^ One study used a flow diagram of patients based on the CONSORT template.^33^ There was no mention of any reporting guideline in the remaining 150 studies.

### Model development and AI architecture

The 148 studies developing or developing and validating models collectively described 276 models (Table 1). Most studies (127/148, 85.8%) worked with 1–3 models. The five external validation-only studies validated five models (one model each).

Nearly 80% of studies (*n* = 120) reported their data pre-processing methods (Table 2). Segmentation was the most used data pre-processing method (*n* = 50/120, 41.7%), followed by data augmentation (*n* = 41/120, 34.2%) and imaging resizing/rescaling (*n* = 26/120, 21.7%).

**Table 2.** Characteristics of the models

Model characteristics	n (%)
**Data pre-processing methods as reported^*a*^**	**120** (**78.4**)
Segmentation (separate background from foreground)^*b*^	50 (41.7)
Data augmentation (image transformation)	39 (32.5)
Resize/scale image	26 (21.7)
Normalise image inputs	24 (20)
Remove noise from image	24 (20)
Marking (separating different objects in the image with markers)	13 (10.8)
Image under- or oversampling	13 (10.8)
Image enhancement	9 (7.5)
Reduce dimensionality	6 (5)
Image reconstruction	5 (4.2)
Image cropping	4 (3.3)
Summarise image inputs	3 (2.5)
Convex hull	3 (2.5)
Bi-cubic Greyscale	2 (1.7) 2 (1.7)
Others	18 (15)
**Model developed**	
Neural network	88 (57.5)
Deep learning	51 (33.3)
Support vector machine	41 (26.8)
Random forest	15 (9.8)
Ensemble methods (multiple algorithms used and combined/‘ensembled’)	13 (8.5)
Decision tree	9 (5.9)
Naïve Bayes	7 (4.6)
Cluster analysis	5 (3.3)
K nearest neighbours	6 (3.9)
Gradient boosting machine	5 (3.3)
Unclear or not well described/named in the paper	5 (3.3)
Other	35 (22.9)
**Unit of analysis reported**	**58** (**37.9**)
Nodules	54 (93.1)
Slice Slice and nodules	2 (3.4) 1 (1.7)
Image annotations	1 (1.7)
**Methods to handle missing data reported**	**13** (**8.5**)
Complete case analysis	12 (92.3)
Other^*b*^	1 (7.7)

SVM, support vector machine.

a More than one alternative possible.

b Authors reported that “support vector machine is able to deal with missing values in training data”, but they did not report how the SVM did this.

Neural networks were most commonly used to develop the models (*n* = 76/276, 27.5%), followed by deep learning (*n* = 47/276, 17%) and support vector machines (*n* = 41/276, 14.9%). The type of model developed was not named or described for five (3.3%) studies (Table 2).

### Sample size

Few articles reported the numbers used in their analyses. Only 58 (38%, Table 2) stated the overall number of patients, images, nodules, or slices that informed their model training or validation. The unit of analysis used was reported in 58 studies (37.9%), of which the nodule was the most common (54; 93.1%). Of the studies working on model development, a minority (*n* = 33/148, 22.3%) reported on the events informing the model training or validation. Table 3 shows the sample sizes used in the studies. A median total of 179 patients (range: 7–62,019) were available per study. The median sample size used for model training was 178 patients (range: 7–59,880). Table 4 shows the sample sizes used in the validation studies.

**Table 3.** Sample sizes informing the analyses in model development studies

*Total*				
	**Patients**	**Images**	**Nodules**	**Slices**
	**Median [IQR], range**	**Median [IQR], range**	**Median [IQR], range**	**Median [IQR], range**
***N***	59	60	46	6
*Total available*	179 [72–569.5], (7–62019)	888 [318–925.8], (20–430067)	1152 [396.8–1360.2], (44–1306040)	27029 [8216–172892], (728–326570)
***N***	48	34	32	4
*Training*	177.5 [77.25–589.75], (7–59880)	888 [119.8–1015], (20–375125)	1091 [676–1219], (94–652612)	56790 [3100–148875], (594–266570)
***N***	16	11	13	2
*Hyperparameter tuning*	72 [49.5–169.2], (10–1113)	888 [118.5–947], (8–47092)	812 [523–2000], (22–140960)	23792 [20688–26896], (17584–30000)
***N***	41	21	26	3
*Internal validation*	178 [80-285], (9–1089)	420 [176-888], (10–7850)	777.5 [133.8–1182.2], (33–139568)	3936 [2035–16968], (134–30000)
***Events***				
***N***	24	5	33	-
*Total available*	158 [74.5–280.2], (26–23132)	5882 [1006–9225], (58–35613)	450 [123–1186], (33–37018)	-
***N***	18	9	22	2
*Training*	79 [52–114.5], (20–12563)	1006 [109–8625], (30–34074)	374.5 [84.5–1050.5], (26–140760)	12329.5 [8684.75–15974.25], (5040–19619)
***N***	8	5	10	2
*Hyperparameter tuning*	167.88 [40.75–156], (2-750)	888 [750–1006], (10–31282)	496.5 [393.8–1538.5], (10–31282)	2126 [1768–2483], (1411–2840)
***N***	21	3	16	2
*Internal validation*	85 [73-179], (8-492)	300 [160–1082], (1864–20)	344 [73.25–462.5], (29612–14)	766 [420–1112], (1458–74)

IQR, interquartile range.

a As 2 papers reported two aims and used different samples for detection and classification, the total is 155 sample sizes.

**Table 4.** Sample sizes informing the analyses in external validation studies

External validation total				
	Patients	Images	Nodules	Slices
	Median [IQR], range	Median [IQR], range	Median [IQR], range	Median [IQR], range
**N** **Total**	10 (13 validation data sets)	7 (9 validations)	5	0
	181 [100–350], (30–1965)	70 [50–346], (5–1015)	56 [39–106], (38–170)	0
**External validation events**				
	Patients	Images	Nodules	Slices
	Median [IQR], range	Median [IQR], range	Median [IQR], range	Median [IQR], range
**N** **Total**	4 (7 validations)	3	5 (7 validations)	0
	111 [75–121], (50–529)	314 [169.5–421.5], (25–529)	83 [37–126], (26–403)	0

*One study reported four external validations and one study reported three validations; total of 22 validation sample sizes.

### Model validation and testing characteristics

Over half of the studies (80/153; 52.2%) compared model results to radiologist evaluations (Table 5). Only eight studies used biopsy results to confirm the diagnosis of lung cancer.

**Table 5.** Model validation methods and performance evaluations

Gold-standard comparator declared	n (%)
Radiologist evaluation^a^	80 (52.3)
Radiologists’ reports available (evaluated retrospectively)	26
Images previously marked/labelled by radiologists	35
Radiologists’ evaluations made for this study (prospectively)	22
No comparison made with a reference standard	28 (18.3)
Another imaging exam	17 (11.1)
Biopsy/histopathology final report	8 (5.2)
Clinical confirmation	1 (0.7)
**Hyperparameter tuning and model internal validation**	
Hyperparameter tuning approach	**50** (**32.7**)
Cross-validation	26 (52)
Split sample	22 (44)
Multisets strategy	1 (2)
Random search	1 (2)
Model internal validation approach^a^	**116** (**75.8**)
Split sample	61 (52.6)
Cross-validation	60 (51.7)
Bootstrapping	3 (2.6)
Training data set	1 (0.9)
**Model performance measures^a^**	
Sensitivity/specificity	86 (56.2)
Accuracy	85 (55.6)
AUC or related measures	68 (44.4)
Precision	14 (9.2)
PPV or NPV	11 (7.2)
F1 score	9 (5.9)
Performance was not evaluated quantitatively	1 (0.7)
Calibration plot or associated measures (*e.g.* slope, intercept)	0 (0)
Other	51 (33.3)
FROC	15
Dice coefficient	11
False positives/scan	7
False-positive rate	6
Youden index	2
CPM	2
Computational time	2

AUC, area under the curve; CPM, competition performance metric; FROC, free-response receiver operating characteristic; NPV, negative predictive value; PPV, positive predictive value.

a More than one alternative possible.

Fifty studies reported their model validation methods, of which cross-validation was most common (*n* = 26/50, 52%). Cross-validation (*n* = 60/116,51.7%) and split-sample (*n* = 61/116, 52.6%) methods were equally commonly used to evaluate the performance of developed models.

Most studies reported diagnostic performance using sensitivity and specificity (86/153; 56.2%), accuracy (percentage of correctly classified; 85/153; 55.6%), or by discrimination values such as the area under the curve (AUC) or with a combination of measures. The AUC was reported in 44.4% of studies (68/153).

Most studies did not mention how the authors dealt with missing data or unreadable images (140/153, 91.5%). 12 studies (7.8%) explicitly reported using only the cases for whom information was complete (*i.e.* discarding the cases with any missing data). In the one remaining study, the authors stated that the support vector machine model they used was able to deal with missing data. However, they did not specify the type of data used (imaging or clinical data) or how they managed missing data.

### Model external validation

A minority of studies (17; 11.1%) carried out external validation. Of these, 12 were model development with external validation studies, two of which have used more than one data set to externally validate the model (one reported four validations and one reported three validations); and five were validation only studies.

Seven of these studies carrying out external validation used publicly available data; with five using LIDC-IDRI and one using LUNA (Supplementary Material 5). One external validation study used a hospital data set, and two used data from the clinical trial NLST (National Lung Screening Trial). Nine of the development with external validation studies reported pre-processing steps, of which seven used segmentation. Three validation-only studies reported pre-processing steps, of which one used image resizing, one used image normalisation, and one used noise removal.

Most of the external validations (13/17; 76.5%) reported predictive performance using sensitivity and specificity, while 10 (58.8%) reported the AUC. Calibration, a key performance measure recommended in the TRIPOD statement, was not assessed in any of the studies.

Table 4 shows that most of the external validation studies (10/17 studies, 58.8%) reported the number of patients, whereas the minority of studies reported the number of events.

### Funding

Most studies were externally funded by organisations other than the academic or research institution where the project was carried out. Of the 153 studies, 103 (67.3%) declared having received some kind of financial support and listed funding sources. They reported one to eight funding organisations each, in one or more grants. No information was available about financial support in 43 of the 153 papers (27.9%). Three (2.9%) studies declared that the study had received no external funding for the research project, while five (4.8%) studies reported support from the academic institution or “self-funded” research. National or regional governments were the most common source of funding (supporting 114 studies). A complete description of the funding sources is available in Supplementary Material 6.

## Discussion

Most of the models included in the 153 studies in our review are still in the development phase, with only five having advanced to the external validation stage; and even those five studies were limited in their study design. The 153 studies reviewed here were poorly reported and lacked standardisation of overall methods, imaging types, pre-processing techniques, and performance metrics. Box 2 indicates some recommendations for developers of diagnostic models for lung cancer (or detection of nodules) using AI methods.

Box 2. **Recommendations for developers of diagnostic models for lung cancer (or detection of nodules) using artificial intelligence methods**

- First, consider validating, updating, and improving models that are already available rather than developing a new model.
- When developing or validating models, report on the study population demographics and clinical characteristics of patients from whom the images were taken.
- Clearly pre-specify study objectives and register protocols with a view to clinical implementation, describing how (and when) the model is expected to be used in clinical practice; report all study results regardless of the model performance.
- Evaluate performance using recommended diagnostic accuracy standards or prediction model metrics.
- When planning model development or validation, select robust gold-standards for comparison. For lung cancer, the best approach is biopsy. When biopsy results are not available, choose a radiology reporting standard or classification system that can be used in subsequent research, allowing comparison between studies. Evaluate interrater agreement.
- When writing up an article on such research, use the most appropriate reporting guidelines available. For diagnostic accuracy studies using artificial intelligence (AI), STARD-AI will be available soon. For reporting comparisons between interventions using AI, use SPIRIT-AI to report your protocol and CONSORT-AI for the final results paper. For prediction models using AI, TRIPOD-AI is under development and will be available soon. Visit the EQUATOR Network website (www.equator-network.org) for further information about reporting guidelines and best reporting practices.

Any claim of effectiveness and safety in healthcare, including methods for the detection of disease, must be supported by robust and transparent evidence.^20^ A model with good diagnostic performance will not necessarily improve outcomes for patients or be clinically relevant or applicable.^20,34^ The, often multiple, models developed in these studies did not test their effectiveness in trials evaluating earlier diagnosis and survival—the patient outcomes that matter for the people who could benefit from earlier detection.

In general prediction research, most studies develop models, few externally validate them, and even fewer evaluate models’ clinical impact.^35^ These issues can be exacerbated by AI technologies where the process of software development does not always follow the established frameworks used in evidence-based medicine, such as validation, clear reporting, and testing, including deployment, usability, and post-market surveillance.^20^

Current regulations for medical devices and general guidelines for the medical use of AI may not be enough to enforce the pre-registration, transparent reporting and code sharing that could allow reproduction and safe application in clinical practice.^36^ Pre-registration requires a study team to publicly prespecify study methodology elements, such as the intended implementation pathway, validation procedures, and power calculations, in study registries or published protocols.^36^ Although mandatory for clinical trials, pre-registration was only widely implemented when medical journals decided only to publish clinical trials that had been pre-registered. However, pre-registration is useful for all study designs, including those developing AI-based models.^12,25,32^

Transparent and complete reporting of methods, results and code would fill the gaps in information^36^ we observed in the studies. The good news is that there is guidance already available for studies using AI. CONSORT-AI for reporting comparisons between interventions using AI, and SPIRIT-AI for their protocols, are tools to guide the reporting of evaluations of the clinical efficacy of interventions based on or including an AI component.^37,38^ STARD-AI is an extension of STARD for diagnostic accuracy studies using AI. The focus of STARD-AI is on the evaluation of AI techniques to assess diagnostic test accuracy and performance in studies using imaging data, pathological data or electronic medical records.^39^ TRIPOD-AI focuses on prediction models that use AI and ML approaches. Both STARD-AI and TRIPOD-AI were under development when this manuscript was submitted for publication.^29,39^ Irrespective of study type, researchers should report thedemographics and clinical characteristics of the participants from whom the images were taken, during both model development and model validation. All study results should be reported, regardless of model performance, to prevent publication bias.

### Diagnostic performance and choice of ground truth

The performance of the reviewed models was generally evaluated using sensitivity, specificity, accuracy, and AUC, with no studies examining calibration. These metrics are commonly used in clinical diagnostic research. However, the metrics of model performance in statistics and AI are often different.^40^ The reviewed studies reported the final performance results but not the data used to calculate them, such as the number of detected nodules andthe total number of patients, or contingency tables, making it difficult for researchers to reproduce study methods.^36^

Two recent reviews of AI models used in lung cancer detection focused on differentiating between benign and malignant nodules, such as indolent and invasive adenocarcinomas. They found highly variable performance results in terms of AUC and low specificity.^19,41^ Another review that focused on studies that used the LIDC-IDRI database found a lack of uniformity in the choice of performance metrics, preventing these models based on the same data set from being compared.^16^ Our results agree with and go beyond the findings of those reviews, as they did not include all types of lung cancers, they did not assess study samples and did not focus on the overall methodology used as we did, they evaluated only the final performance of the model.

Model performance should be assessed against a ground-truth or ‘gold-standard’ definition of lung cancer and should be defined before model development. Although the often-accepted gold-standard for cancer diagnosis is a positive biopsy result,^10^ biopsies can be highly invasive and are not always possible. We found that less than 6% of reviewed studies compared the performance of AI models to biopsy results.The majority (52.3%) compared their AI algorithm to radiologists’ reports or annotations that were available in public data sets of chest CT images available online—although, according to the WHO, “*annotations alone are insufficient as ground truth where a biopsy or pathologic investigation is needed to confirm the prediction/diagnosis*.”^20^ A possible reason for this choice is that most studies used patient databases that are publicly available, without the biopsy results that would be available from hospital cohorts.

Free, easy access to public data sets of chest CT images has facilitated the development of many of the reviewed models. Almost half of the included studies (49%) used LIDC-IDRI, or LUNA, or a combination of these two CT scan data sets. Public image data sets often provide scans of lung nodules marked by radiologists (three, four or more) to localise lesions of interest or classify them as benign or malignant. Although these databases hold a large amount of data, there is limited information about the data quality. Differences in the training radiologists receive in low- or high-income countries^20^ may affect the number of cases considered suspicious, the uniformity of the criteria for “positive” cases^40^ and therefore model comparability.

The studies included in our review used different, often imprecise, definitions of “positive cases” of lung cancer, such as “nodules classified as malignant tumours” or “any detected nodule, whether benign or malignant.” The reference standard for a nodule also varied, even between studies using the same databases. Some studies accepted any nodule identified by one radiologist, others only included nodules identified by two or three radiologists, and still others required both identification from multiple radiologists and a biopsy result as ground truth—in other words, the “correct answers” needed to train the models.^40^ Currently, lung-RADS recommends the radiological reporting of “the most concerning feature”, that can be a solid or non-solid lesion or even an association between both.^5^ However, Lung-RADS was not available when the included studies were being conducted. The vast differences between the studies mean that diagnostic performance and claims about model usefulness^15^ cannot be compared.^13,15,16,24^

Even the studies using more than one radiologist evaluation did not use a standardised imaging diagnostic system and did not report on measures such as interobserver agreement. Hopefully, the CT databases will incorporate an imaging standard for case description in the future. The recently published lung-RADS (Lung CT Screening Reporting and Data System), by the American College of Radiology,^4^ might be a solution.

### Studies focus on technology, not patients

The reviewed studies often failed to report demographic information about the patients whose images were used, such as age and sex. Data that would be relevant for lung cancer, such as tobacco use and clinical history of respiratory and other non-malignant lung diseases, were also rarely reported, although this information may not have been available in public data sets. It was therefore impossible to verify whether the patient samples used were representative of the models’ target populations. The characteristics of the patient sample used are crucial for study reproducibility,^20,36^ yet the background information on the humans behind the images was not reported in many of these studies.

The issue of missing clinical and demographic datawas seen both in studies using publicly available data sets and studies using local hospital data sources. The use of public databases certainly facilitated model development and training, but studies using hospital-based data also failed to share data or provide patients’ characteristics (*e.g.* age, smoking status) that would be necessary to predict malignancy risk. We believe that the adoption of the lung-RADS 2022 system^5^ for the radiologic report of CT screening may help standardise the categorisation of the lesions and the final radiologic diagnostic report, allowing comparisons between studies with different samples. However, the importance of including details about the patient remains, as emphasised by lung-RADS, which requires information on prior exams and suggests follow-up for certain categories. Ultimately, biopsy remains the gold-standard to determine if the nodule is cancerous.

Most studies were conducted on public databases of CT scans, whose diagnostic performance cannot be compared with those using X-rays. The studies also did not report information about the equipment used to acquire images, which is necessary to evaluate whether the quality of images varies in other regions or settings.^17,19,34^ The quality of equipment can affect the ability of the radiologists or the AI systems to identify lung nodules.

As model development and analysis were usually based on images alone, the papers usually started by giving the sample sizes as “cases” or “scans” of individuals who underwent lung examination. However, they then reported model performance using a different unit of analysis, such as imaging slices, or patches of images, and not always “nodules”. And even detecting nodules is not the same as detecting cancer—as around 1 in 20 nodules detected can be diagnosed as malignant.^1^ The number of images, scans, or slices used for each patient was often unclear.

As studies used different denominators (*i.e.* patients, scans, or images), it was difficult to evaluate whether sample sizes were adequate for detecting events, which could be nodules, malignant tumours, or correctly segmented nodules. Studies did not justify the sample sizes used, with most using convenience samples of “all available images” in their data set or at their hospital. A review of the use of AI in prediction models across all oncology found the same result.^12^ The authors also found that studies often failed to discuss missing data, which for studies in imaging can be missing slices, lost or corrupted image files, or clinical data.^12^

This review has some limitations. We only evaluated papers published in 2018 and 2019, and although this provided a contemporary sample of studies at the time of the search, some eligible studies may have been missed. We also did not have the resources to translate and evaluate studies published in non-English languages. However, our aim was to report the characteristics of recent studies to reflect current research practice, and it is unlikely that additional studies (including studies only available in non-English languages) would change the conclusions of this review.

Subsequent studies may now also be available that externally validate AI models that were developed in the studies included in our review, and it would be useful to update ourreview of the research landscape to include these studies and ongoing research in the area. Future studies should observe recommendations such as those listed in Box 2.

## Conclusions

The methods used to develop, validate, and test cancer prediction models that use AI to detect, segment, or classify pulmonary nodules as benign or malignant in medical imaging vary, are poorly reported and are difficult to evaluate. Comparing the performance between models is challenging and cannot be done as diagnostic performance is often assessed using several different metrics and units of measurement. Studies developing or validating AI models cannot be easily replicated as important information about patient characteristics is not reported.
